# Supplementary material for: Empirical methods for controlling false positives and estimating confidence in ChIP-Seq peaks
Source: BMC Bioinformatics. 2008 Dec 5;9:523. doi: 10.1186/1471-2105-9-523 (PMC2628906; doi:10.1186/1471-2105-9-523)
Supplement: Additional File 1 — A variety of html documents from the USeq web site detailing the available applications, their best usage, output file type descriptions, command line menus, etc. [file 1471-2105-9-523-S1.zip › USeqUserGuides/JavaDoc.zip!/JavaDoc/gata/plotter/Sliders.html]

Sliders


|  |  |  |
| --- | --- | --- |
| **Overview** \| **Package** \| **Class** \| **Use** \| **Tree** \| **Deprecated** \| **Index** \| **Help** | |  |
| **PREV CLASS**   **NEXT CLASS** | **FRAMES**    **NO FRAMES**     **All Classes** |  |
| SUMMARY: NESTED \| FIELD \| CONSTR \| METHOD | DETAIL: FIELD \| CONSTR \| METHOD |  |


---


## gata.plotter Class Sliders

```
java.lang.Object
  gata.plotter.Sliders
```

---

``` public class Sliders extends java.lang.Object ```

**Author:**
:   nix
    Class representing the sliders used to show or hide box-line-box shapes in the DNA alignment

---

**Constructor Summary**

`Sliders(AlignParams ap,
GATAParams params)`


| **Method Summary** | |
| --- | --- |
| `javax.swing.JSlider[]` | `getSliders()` |
| `void` | `makeSlider(javax.swing.JSlider s, int major, int minor)` |

**Methods inherited from class java.lang.Object**

`equals, getClass, hashCode, notify, notifyAll, toString, wait, wait, wait`

**Constructor Detail**

### Sliders

```
public Sliders(AlignParams ap,
               GATAParams params)
```


**Method Detail**

### getSliders

```
public javax.swing.JSlider[] getSliders()
```

---


### makeSlider

```
public void makeSlider(javax.swing.JSlider s,
                       int major,
                       int minor)
```


---


|  |  |  |
| --- | --- | --- |
| **Overview** \| **Package** \| **Class** \| **Use** \| **Tree** \| **Deprecated** \| **Index** \| **Help** | |  |
| **PREV CLASS**   **NEXT CLASS** | **FRAMES**    **NO FRAMES**     **All Classes** |  |
| SUMMARY: NESTED \| FIELD \| CONSTR \| METHOD | DETAIL: FIELD \| CONSTR \| METHOD |  |


---
